# Set targets, assess response, intervene early to achieve minimal disease activity in atopic dermatitis (STRIVE AD): A modified Delphi consensus project to optimize management of atopic dermatitis in the United Kingdom

**DOI:** 10.1016/j.jdin.2025.11.028

**Published:** 2025-12-17

**Authors:** Maria Angeliki Gkini, Helen Alexander, Maria Barfoot, Alberto Barea, Paula E. Beattie, Firas C. Kreeshan, Deborah Moffitt, Padma Mohandas, Andrew E. Pink, Andrew Proctor, Ann Sergeant, Laura J. Savage

**Affiliations:** a Department of Dermatology, Barts Health NHS Trust, London, United Kingdom; b Department of Dermatology, St John’s Institute of Dermatology, Guy’s & St Thomas’ NHS Foundation Trust and King’s College London, London, United Kingdom; c Department of Dermatology, Gloucestershire Hospitals NHS Foundation Trust, Cheltenham, United Kingdom; d Department of Dermatology, Kingston Hospital NHS Foundation Trust, Kingston upon Thames, United Kingdom; e Department of Dermatology, NHS Greater Glasgow and Clyde, Glasgow, Scotland; f Department of Dermatology, Salford Royal NHS Foundation Trust, Salford, United Kingdom; g National Eczema Society, London, United Kingdom; h Department of Dermatology, NHS Fife, Kirkcaldy, Scotland; i Department of Dermatology, Leeds Teaching Hospitals NHS Trust and University of Leeds, Leeds, England

**Keywords:** Atopic dermatitis, consensus recommendations, Delphi, eczema, guidelines, minimal disease activity, treatment targets, United Kingdom

## Abstract

**Background:**

Guidelines for the management and treatment of atopic dermatitis have been extensively published in Europe and globally, however these are not always relevant to the United Kingdom (UK) clinical setting.

**Objectives:**

To develop consensus statements for the optimized management of adults and children with atopic dermatitis in the UK.

**Method:**

Consensus statements were developed by an expert advisory panel following a targeted literature review, based on 5 topics (patient centric approach, assessing severity consistently, setting treatment targets, early intervention, and optimizing treatment). Consensus statements were voted on using a 5-point Likert scale in a Delphi over 3 rounds by 12 multidisciplinary, geographically spread participants. Consensus was reached if ≥75% of the voting participants rated the statement as 4 or 5.

**Results:**

Strong consensus (≥90%) was achieved for 24 out of 27 statements. The level of consensus by topic was: Patient centric approach (92%-100%); Assessing severity consistently (75%-100%); Setting treatment targets (83%-100%); Intervening early (92%-100%); Optimizing treatment (92%).

**Limitations:**

Due to the design and objectives of the study only a small number of participants were invited to take part.

**Conclusions:**

The following recommendations have been developed to be practical and easy to implement within everyday clinical practice.

**Capsule Summary**

• Current European/global guidelines for the treatment/management of atopic dermatitis are not always applicable based on specific challenges within UK-based clinical practice.
• Minimal disease activity (eczema area and severity index-90, Itch numeric rating scale-0-1) and recommendations in this paper are raising the bar for standard of UK care in atopic dermatitis.

## Introduction

Atopic dermatitis (AD) is a chronic inflammatory disorder characterized by dysfunction of the skin barrier, leading to erythema, scale, lichenification, and edema, upregulation of T-helper type 2-mediated immune responses and pruritus.^1^ In a recent National Institute for Health and Care Excellence (NICE) appraisal, patients and professional organizations described AD as “life-limiting, debilitating, and isolating, and affecting all aspects of life.” In particular, severe disease was associated with intolerable pruritus that disrupts sleep, leading to a higher risk of depression and suicide,^2^ with increased health-related and socioeconomic burdens.^1^

Standard treatment options for moderate-to-severe AD include systemic immunosuppressants (methotrexate, ciclosporin, azathioprine, or mycophenolate mofetil),^3^ targeted immunomodulators include biologics (dupilumab, tralokinumab, lebrikizumab, nemolizumab), and janus kinase inhibitors (JAKi) (baricitinib, upadacitinib, abrocitinib), which can be used in patients where the disease has not responded to, or there is a contraindicated to, at least 1 systemic immunosuppressant and/or phototherapy.^2^^,^^4^, ^5^, ^6^, ^7^

Although numerous guidelines and consensus statements exist at a global and European level (Table I), the recommendations for identifying and managing patients who need systemic treatment are inconsistent.^8^^,^^10^^,^^11^^,^^14^, ^15^, ^16^, ^17^, ^18^, ^19^ Recent guidelines have focused on treat-to-target recommendations for AD management, including utilizing clinician (Eczema Area and Severity Index [EASI]: 75; SCORing Atopic Dermatitis [SCORAD]: 75 or SCORAD ≤ 24; absolute peak/worst pruritus Numerical Rating Score [NRS] ≤ 4) and patient (Dermatology Life Quality Index [DLQI] ≤ 5; Patient Oriented Eczema Measure [POEM] ≤ 7) reported outcome measures as treatment targets at 6 months.^20^ Furthermore, Yeung *et al*^21^ proposed higher treatment target of EASI90 at 1 year and there have also been suggestions of a combined treat-to-target and shared decision-making initiative.^22^ Currently, there are no United Kingdom (UK)-specific guidelines for the treatment and management of AD in adults and adolescents, and global and European guidelines are not always applicable to the UK-based clinical practice, due to the strict restrictions at a national (NICE) and local (individual hospitals) level, which may limit treatment options based on finances and severity of disease.

**Table I:** Current guidelines for the diagnosis and treatment of moderate-to-severe atopic dermatitis globally, in Europe and the United Kingdom

	Global^8^^,^^9^	Europe^10^^,^^11^	Current NICE position^3^^,^^12^^,^^13^
**Diagnosis**	Take comprehensive patient history Visually assess skin condition	Take comprehensive patient history Visually assess skin condition	Take comprehensive clinical and drug history Visually assess skin condition
**Assessing disease severity**	Assess skin severity Assess psychological/mental health (QoL)	Assess objective and subjective symptoms consistently	Assess skin and physical severity, Assess impact of QoL and psychosocial wellbeing
Patient reported outcome	POEM, PO-SCORAD, (NRS)-itch, DLQI, CDLQI, EuroQoL-5D or Short-Form 36	PO-SCORAD DLQI, POEM	POEM, CDLQI, DLQI
Clinician reported outcome	IGA, EASI and SCORAD	SCORAD, EASI	VAS, EASI, IGA, PGA
Flare avoidance strategies	Assess triggers associated with the body and environmental factors	Allergy workup (Serum IgE, Skin prick test, Patch test), avoidance of allergens, educational programs	Assess physical and environmental triggers, test for allergens where indicated (Patch or radioallergosorbent test IgE for substances), dietary modification where clinically indicated
Treatment approach	Stepwise approach	Stepwise approach	Stepwise approach
**Treatment for adults**			
Moderate	Topical corticosteroids, topical calcineurin inhibitors, phototherapy	Topical calcineurin inhibitors or topical corticosteroids, wet wrap therapy, Narrow band ultraviolet B and medium dose ultraviolet A1, psychosomatic counselling	Emollients, moderate-potency topical corticosteroids, topical calcineurin inhibitors, bandages
Severe	Conventional and new systemic therapies	Conventional systemic drugs: ciclosporin, azathioprine, methotrexate and systemic glucocorticosteroids; Biologics: Dupilumab, lebrikizumab, tralokinumab; JAKI: Abrocitinib, baricitinib, upadacitinib	Emollients, potent topical corticosteroids, topical calcineurin inhibitors, bandages, phototherapy, oral corticosteroids, systemic therapy
**Treatment for children**			
Moderate	Emollients, topical steroids, topical calcineurin inhibitors	Topical calcineurin inhibitors or topical corticosteroids, wet wrap therapy, Narrow band ultraviolet B and medium dose ultraviolet A1, psychosomatic counselling	Emollients, moderate-potency topical corticosteroids, topical calcineurin inhibitors, bandages
Severe	Emollients, topical steroids, topical calcineurin inhibitors, phototherapy, systemic immunosuppressants	Conventional systemic drugs: ciclosporin (≥16 y), azathioprine, methotrexate; Biologics: Dupilumab (≥6 mo), lebrikizumab (≥12 y), tralokinumab (≥12 y); JAKI: Abrocitinib (≥12 y), baricitinib (≥2 y), Upadacitinib (≥12 y)	Emollients, potent topical corticosteroids, topical calcineurin inhibitors, bandages, Phototherapy, systemic therapy
**Optimal treatment target**	EASI-75, SCORAD75	EASI-75, SCORAD75	EASI-50
Achieved by	3-6 mo	16 wk	16 wk

*CDLQI*, Children's Dermatology Life Quality Index; *DLQI*, Dermatology Life Quality Index; *EASI*, Eczema Area and Severity Index; *IGA*, Investigator Global Assessment; *IgE*, Immunoglobulin E; *JAKI*, janus kinase inhibitors; *NRS*, Numeric Rating Scale; *PGA*, physicians’ global assessment; *POEM*, Patient-Oriented Eczema Measure; *PO-SCORAD*, Patient-Oriented SCORAD; *QoL*, quality of life; *SCORAD*, SCORing Atopic Dermatitis; *UK*, United Kingdom; *VAS*, Visual Analogue Scale.

The Delphi process is a widely accepted method in healthcare for determining consensus, due to its ability to allow equal decision-making, anonymity, rapid response, and the effective gathering of opinions from multiple clinicians from diverse geographical locations through feedback.^23^^,^^24^ Delphi studies use open-ended questions to develop consensus over several rounds using feedback from prior rounds. While modified Delphi studies use predefined statements that are refined by feedback over several rounds.^23^ Based on these considerations, the aim of this study was to develop a set of guiding principles underpinned by clinical consensus, which can be used to optimize the management of patients with AD in the UK, using a modified Delphi process with 3 rounds and 1 interim virtual meeting.

## Materials and methods

### Consensus statement development

**Fig 1:**
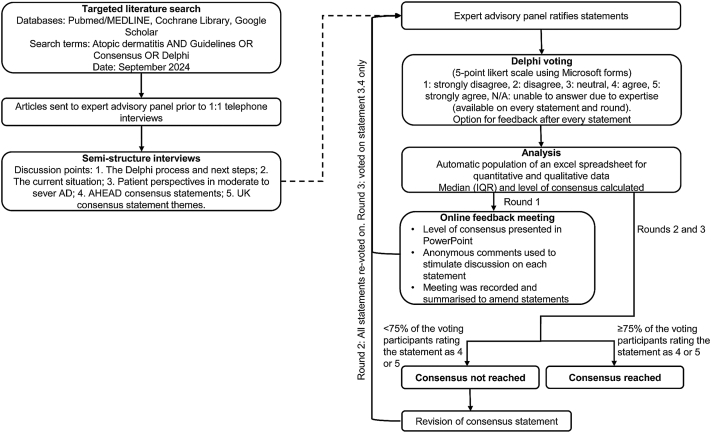
Atopic dermatitis: Modified Delphi methodology to build consensus in the treatment and management of patients with atopic dermatitis within the UK. *AD*, Atopic dermatitis; *IQR*, interquartile range; *N*/A, not applicable; *UK*, United Kingdom.

An initial literature search was conducted (September 2024) to identify current guidelines for the management of patients with AD globally, and to identify research gaps relating to the UK. Searches were performed using PubMed/MEDLINE, Cochrane Library and Google Scholar. Search terms included: “atopic dermatitis” AND “guidelines” OR “consensus” OR “Delphi,” with particular focus on papers relating to treat-to-target. In total 11 papers,^15^, ^16^, ^17^^,^^20^^,^^22^^,^^25^, ^26^, ^27^, ^28^, ^29^, ^30^ were obtained and were sent to the expert advisory panel (2 experienced consultant medical dermatologists [MAG and LJS] and 1 patient representative from an eczema charity [AP]) prior to a series of initial 1-hour semistructured 1:1 telephone interview performed in October 2024. Based on the literature search and interviews, a list of consensus statements were developed and ratified by the clinicians of the expert advisory panel based on 5 broad areas of interest: taking a patient centric approach, assessing severity consistently, setting treatment targets, early intervention and optimizing treatment. The final consensus statements after each round were ratified by the expert advisory panel clinicians before re-voting by the participants. The modified Delphi process is outlined in Fig 1.

### Participant selection

UK-based healthcare professionals (HCPs) and 1 eczema charity representative, were selected from UK participants of the AHEAD study,^22^ lists of ‘jobbing’ dermatologists and peer recommendations. Participants included the 3 members of the expert advisory panel. Inclusion criteria required that participants had expert knowledge and qualification in dermatology; experience in the management of patients with AD in the UK and interest in optimizing care of patients with AD. To provide a UK wide perspective, experts were also identified based on geographical location and treatment settings including secondary and tertiary care. Participants received financial recompense for their time.

### Delphi vote

A maximum of 3 rounds of voting was conducted, with voting on all statements conducted in rounds 1 and 2 and only statements not reaching consensus in round 3. Each round participants were emailed a link to Microsoft forms and were required to complete the questionnaire over 1 week. The questionnaire included 27 statements and participants were required to rate the agreement on each statement using a 5-point Likert scale (1 = strongly disagree; 2 = disagree, 3 = neutral, 4 = agree, 5 = strongly agree). A “not applicable” option was included for each statement to allow the participants to abstain from voting on any statements, which were outside their field of expertise along with a comment box to provide feedback on any statements. Anonymity was maintained throughout the voting process and all responses were given equal voting power.

### Statistical analysis

Qualitative and quantitative data generated by Microsoft forms was used to automatically populate Excel. Descriptive statistics including, median (interquartile range), level of consensus (percentage) and interrater agreement between voting rounds (percentage) were calculated. Consensus was predefined as ≥75% of the voting participants rating the statement as 4 or 5 (agree or strongly agree), whereas strong consensus was defined as ≥90% agreement by the voting participants. A <5% change in responses between rounds was used as the criterion for stability and to determine when further consultation was unnecessary.

### Online feedback meeting

Results from the first round of voting were combined and presented in PowerPoint during an online group meeting. All statements including those which did reach consensus were discussed by theme using anonymous comments generated during the first round Delphi vote to stimulate discussion. The online meeting was recorded and summarized. Feedback on the consensus statements from the round 1 Delphi vote and the online meeting were used to revise the consensus statements to be used in round 2.

## Results

### Participants

In all, 16 participants were invited to take part in the study (Table II), with 12 participants accepting the invitation. The participants consisted of 8 (66.7%) consultant dermatologists, 1 (8.3%) dermatology research registrar, 2 (16.7%) nurse specialists, and 1 (8.3%) eczema charity representative. Four (36.4%) participants practice in a secondary treatment setting while 7 (63.6%) practice in a tertiary treatment setting.

**Table II:** Participant demographics

Sample frame	UK participants of the AHEAD study,^22^ Lists of “jobbing” dermatologists (nurses/doctors/patient advocacy representatives) from tertiary and secondary care facilities, peer recommendations	
Number of invites sent	16	
Acceptances	12	
Rejections	4∗	
**Participants**	***N* = 12**	**Percentage of participants(%)**
**Geography**		
London	4	33.3
South of England	3	25
North of England	2	16.7
Scotland	2	16.7
Nationwide	1	8.3
**Treatment setting**	***N* = 11**	
Secondary	4	36.4
Tertiary	7	63.6
**Role/expertise**	***N* = 12**	
Consultant dermatologist	8	66.7
Dermatology research registrar	1	8.3
Nurse specialist	2	16.7
Eczema charity representative	1	8.3

∗ Participants declined as they could not attend the timed meetings specified.

### Delphi vote

**Fig 2:**
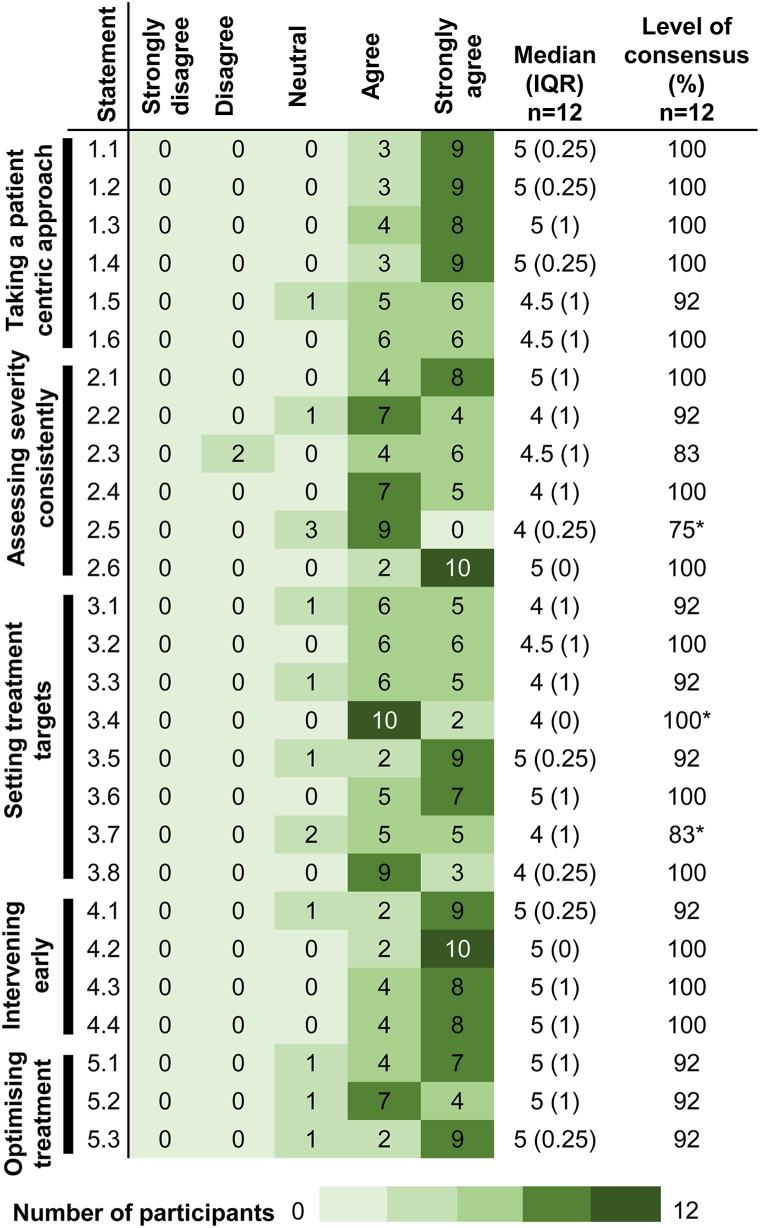
Atopic dermatitis. Heatmap representing the final Delphi consensus process for the treatment and management of patients with atopic dermatitis. ∗Indicates statements which did not reach consensus during round 1 and 2. *IQR*, Interquartile range.

A total of 27 statements based on 5 topics were reviewed, presented and voted on up to 3 Delphi rounds. In rounds 1 (conducted November 2024) and 2 (conducted December 2024), a total of 11 and 12 participants responded (representing 91.7% and 100% of the participants) respectively. Consensus was reached for 24 statements in round 1 and 26 statements in round 2, with an aggregate agreement of between 82% to 100% in round 1 and 75% to 100% in round 2 (Table III). Three statements did not reach consensus after round 1 (statements 2.5, 3.4, and 3.7) with a 64% to 73% level of consensus. Only 1 statement did not reach consensus after round 2 (Statement 3.4) with a 67% level of consensus. Re-voting was performed following revision (language framing) of these proposed statements. In Delphi round 3 (conducted January 2025), 12 (100%) participants responded, with the final remaining statement after revisions reaching 100% consensus A strong consensus (92%-100%) was reached for 24 out of the final 27 statements. Between rounds 1 and 2, 67% of participants did not change their response, whereas 97% of participants did not change their responses between rounds 2 and 3 (for those repeated items), indicating a high degree of consistency in participant opinions. The 27 consensus-based recommendations focused on taking a patient centric approach (92%-100% consensus), assessing severity consistently (75%-100% consensus), setting treatment targets (83%-100% consensus), early intervention (92%-100% consensus) and optimizing treatment (92% consensus) in relation to UK National Health Service (NHS) practice. The level of consensus for the final agreed statements can be found in Fig 2.

**Table III:** Level of consensus from voting rounds 1 to 3

Statement	Voting round	Number of participants *n*	Strongly disagree	Disagree	Neutral	Agree	Strongly agree	Median (IQR)	Level of consensus (%)
1.1	1	11	0	0	1	4	6	5 (1)	91
	2	12	0	0	0	3	9	5 (0.25)	100
1.2	1	11	0	0	0	2	9	5 (0)	100
	2	12	0	0	0	3	9	5 (0.25)	100
1.3	1	11	0	0	0	2	9	5 (0)	100
	2	12	0	0	0	4	8	5 (1)	100
1.4	1	11	0	0	0	4	7	5 (1)	100
	2	12	0	0	0	3	9	5 (0.25)	100
1.5	1	11	0	0	0	5	6	5 (1)	100
	2	12	0	0	1	5	6	4.5 (1)	92
1.6	1	11	0	0	1	5	5	5 (1)	91
	2	12	0	0	0	6	6	4.5 (1)	100
2.1	1	11	0	0	0	5	6	5 (1)	100
	2	12	0	0	0	4	8	5 (1)	100
2.2	1	11	0	0	1	4	6	5 (1)	91
	2	12	0	0	1	7	4	4 (1)	92
2.3	1	11	0	1	0	4	6	5 (1)	91
	2	12	0	2	0	4	6	4.5 (1)	83
2.4	1	11	0	0	0	6	5	4 (1)	100
	2	12	0	0	0	7	5	4 (1)	100
2.5	1	11	0	1	2	6	2	4 (0.5)	73
	2	12	0	0	3	9	0	4 (0.25)	75
2.6	1	11	0	0	0	3	8	5 (0.5)	100
	2	12	0	0	0	2	10	5 (0)	100
3.1	1	11	0	1	0	5	5	4 (1)	91
	2	12	0	0	1	6	5	4 (1)	92
3.2	1	11	0	0	1	5	5	4 (1)	91
	2	12	0	0	1	6	5	4.5 (1)	92
	3	12	0	0	0	6	6	4.5 (1)	100
3.3	1	11	0	0	0	4	7	5 (1)	100
	2	12	0	0	1	6	5	4 (1)	100
3.4	1	11	0	1	3	1	6	5 (2)	64
	2	12	0	1	3	3	5	4 (2)	67
	3	12	0	0	0	10	2	4 (0)	100
3.5	1	11	0	0	1	1	9	5 (0)	91
	2	12	0	0	1	2	9	5 (0.25)	92
3.6	1	11	0	0	2	4	5	4 (1)	82
	2	12	0	0	0	5	7	5 (1)	100
3.7	1	11	0	0	3	5	3	4 (1)	73
	2	12	0	0	2	5	5	4 (1)	83
3.8	1	11	0	0	2	4	5	4 (1)	82
	2	12	0	0	0	9	3	4 (0.25)	100
4.1	1	11	0	0	0	3	8	5 (0.5)	100
	2	12	0	0	1	2	9	5 (0.25)	92
4.2	1	11	0	0	1	1	9	5 (0)	91
	2	12	0	0	0	2	10	5 (0)	100
4.3	1	11	0	0	0	4	7	5 (1)	100
	2	12	0	0	0	4	8	5 (1)	100
4.4	1	11	0	0	0	6	5	4 (1)	100
	2	12	0	0	0	4	8	5 (1)	100
5.1	1	11	0	0	0	5	6	5 (1)	100
	2	12	0	0	1	4	7	5 (1)	92
5.2	1	11	0	0	0	5	6	5 (1)	100
	2	12	0	0	1	7	4	5 (1)	92
5.3	1	11	0	0	0	3	8	5 (0.5)	100
	2	12	0	0	1	2	9	5 (0.25)	92

### Narrative feedback

Participants were invited to provide feedback on every statement in the Delphi vote and during the open feedback meeting. Theme-specific feedback resulted in the revised wording of 11 of the statements after round 1, even though 9 of those statements had reached consensus after round 1 (Table IV). The general comments for the 3 statements not reaching consensus were as follows: Statement 2.5: *Panelists agreed that apps are useful tools for calculating disease severity efficiently, especially the recording of information around flares; however, it was agreed that this would not necessarily reduce the burden to the patient or the NHS*. Statement 3.4: *It was felt that some systemic treatments take time to begin working and concern was raised around switching patients between treatments too quickly, given the limited treatment options in the most severe patients. For these reasons, it was suggested that realistic improvements be added to the statement for round 2 and (where possible) be added to the statement for round 3.* Statement 3.7*: There was acceptance between participants that for some patients, minimal disease activity (MDA) may not be achievable; however, the panel agreed that with the advent of new efficacious treatments and several in phase III clinical trial and the implementation of patient centered treatment plans; HCPs should strive toward reaching these targets, for these reasons the wording of this statement was not amended for round 2*.

**Table IV:** Final consensus statements with discussion themes and modifications to consensus wording from round 1

Final consensus statement	Discussion themes
***Theme 1: Patient centric approach***	
1.1: HCPs should seek to understand the most burdensome aspects of the condition for the patient by asking open questions such as (“what would your life be like without your skin condition) **“How is this condition impacting you? What is it stopping you doing?”**	Whilst “what could your life be like without your skin condition” is very impactful the panel felt this could upset patients. Open questions should focus on the patient now and not a perceived future state.
1.2 With the growing number of treatment options, HCPs should communicate what they hope to achieve in terms of improvement in skin and the reduction of symptoms such as pruritus, pain and sleeplessness.	
1.3 Treatment decisions should be made and targets should be set as part of a shared decision-making process and should be (based on a combination of patient and clinician reported outcomes. Patient preference should also be considered.) **multifaceted, holistic and patient specific, taking into account patient expectations and what the HCPs believe is achievable**.	All patients are different and many have complex needs. Expectations vary widely with some patients willing to accept small improvements in quality of life that others would not. It is important to convey to patients what is possible without falsely raising hopes. This needs to be done as part of a conversation and not solely based on outcome measures.
1.4 Treatment decisions should take into account the multifaceted burden of AD on patients.	
1.5 Where available locally, HCPs should consider ways to aid communications, patient empowerment and satisfaction (medical photography, information leaflets, screening tools).	
1.6 HCPs should discuss with the patient/caregiver the results of any outcome measures used and explain what the results mean in terms of disease severity and **how they may be used, in conjunction with other factors, when considering treatment choices.**	The panel were keen that decisions on treatment were not solely based on outcome measures, but that outcomes measures are considered in conjunction with other factors such as treatment history, patient characteristics and needs.
***Theme 2: Assessing severity consistently***	
2.1: HCP and PRO measures are a very important aspect of assessing severity of disease and response to treatment in AD and aiding communication between HCPs.	
2.2: As a minimum PROs including a NRS for pruritus and DLQI and the CRO EASI should be used to inform treatment decisions.	Many scales are available, however the panel agreed that EASI and DLQI are widely used in the UK and align with NICE requirements. Including a simple scale for pruritus is also important as this is 1 of the most troublesome symptoms for patients and is poorly covered by DLQI.
2.3: Disease severity should be measured consistently every time the patient is seen in the clinic and documented in an accessible place in the patient’s medical records to provide robust information about treatment response.	There was discussion about whether this is practical and necessary in patients who are stable on long term treatments. On balance, the panel agreed that given NHS structure and working practices, this is important.
2.4: HCPs should assess presence and severity of any psychiatric and psychosocial conditions associated with AD by consistent use of standardized measures and refer patients promptly as necessary.	
2.5: Digital innovations and technologies, such as apps should be considered for measuring disease severity and frequency of flares, where these are available (and could reduce the burden to the patient and the NHS.)	The panel considered that digital technologies such as apps have a particular place in measuring disease severity and are especially useful for patients in recording information about flares in their condition. Reducing burden to patient and NHS was removed as the panel considered that often, while important, they can actually add burden.
2.6: Time should be allowed for patients to complete PROs before the appointment to maximize the time available with the HCP.	
***Theme 3: Setting treatment targets***	
3.1: The ultimate treatment target for HCPs in AD should be a (satisfied) patient with clear/almost-clear skin with no/minimal pruritus.	Panelists felt that while a patient may have clear skin they may not be satisfied due to psychiatric and psychosocial comorbidities. Conversely, some patients with poor disease control may be satisfied because their expectations of treatment are low and this should not preclude them receiving more efficacious treatments as these become available.
3.2: When setting targets, HCPs should consider immediate (within 4 wk), short-term (3-4mo) (**3-6 mo)** and long term (1 y and beyond) goals for individual patients.	The panel recommended to expand the timeframe considered “short term” to allow more time before assessing short-term response and to allow more flexibility and mirror NHS practice in terms of clinic schedules.
3.3: Immediate goals should focus on providing relief from symptoms such as pruritus and pain.	
3.4: Short-term goals should focus on clear or almost-clear skin, (significant)**(wherever possible) realistic** improvement in symptoms such as pruritus and (redness and) improvements in quality of life.	The panel discussed that clear or almost-clear skin is not possible in every patient, even with more advanced treatments now available. There was also concern about switching patients between treatments too quickly, given limited treatment options in the most severe patients.
3.5: Long term goals should focus on sustained disease control, reduction in the number of flares, use of topical steroids and management of AD related psychiatric and psychosocial comorbidities.	
3.6: Targets should be stretching and set with patients to take into account the patient goals at each stage.	
3.7: Based on availability of more effective therapies, HCPs should aspire to reach targets of EASI 90 or EASI ≤3, NRS for pruritus of ≤1, DLQI ≤3 4-6 mo after starting a new treatment.	There is acceptance that for some patients, MDA may not be achievable, however the panel agreed that with the advent of new efficacious treatments and several in phase III clinical trial and the implementation of patient centered treatment plans, HCPs should strive towards reaching these targets.
3.8: A treat-to-target approach should be employed with response to treatment being assessed regularly and modifications to treatment made to get the patient as close to target as considered possible.	
***Theme 4: Intervening early***	
4.1: The effect of AD on a patient's life course is well documented and as such HCPs should intervene at the earliest opportunity to ensure optimal treatment is offered as soon as possible.	
4.2: Intervening early is especially important in younger patients (adolescents and young adults) since there is more scope to minimize the impact on life course. Systemic and targeted therapy should also be considered in this group if and when appropriate.	The panel did not consider it necessary to specify adolescents and young adults.
4.3: It is important to raise awareness amongst Primary Care HCPs of the (new treatment)**advanced therapy treatments**, which are available and to support them to identify and refer appropriate patients to secondary care where topical treatment have not been effective so that other treatments can be initiated quickly.	Wording amended slightly to reflect that primary HCP’s should be aware of all advanced treatment options and not just new ones.
4.4: There is significant (NHS)**healthcare** resource and patient financial burden/indirect costs associated with AD that is not optimally controlled as such HCPs should aim to follow GIRFT principles to aim to treat with the most appropriate treatment.	
***Theme 5: Optimizing treatment***	
5.1: HCPs and patients should aim for (optimum disease control) MDA (defined as clear/almost-clear skin with no/minimal pruritus).	Panel advice to use 1 term consistently. MDA was favored as this is used in other areas of immunology.
5.2: **Across the treatment spectrum, HCPs** (HCPs should review whether optimum disease control) should review whether MDA has been reached for an individual patient based on their goals at every review and where a patient has failed to reach this control, consideration should be given to optimization of treatment to achieve tighter control.	Some panelists felt that medication safety profile may also be a factor in optimizing treatment.
5.3: Systemic and targeted therapy should be considered in patients with moderate-to-severe AD who have failed to achieve the agreed targets with topical medications or phototherapy, in line with relevant NICE guidance.	

Wording removed from the original consensus statements are (underlined). Wording added to the original consensus statements are in **bold**.

*AD*, Atopic dermatitis; *CRO*, clinician reported outcome; *DLQI*, Dermatology life quality index; *EASI*, Eczema Area and Severity Index; *GIRFT*, get it right first time; *HCP*, health care professional; *MDA*, minimal disease activity; *NHS*, National Health Service; *NICE*, National Institute for Health and Care Excellence; *NRS*, numerical rating scale; *PRO*, patient reported outcome.

## Discussion

In the absence of recent national UK guidance for the treatment and management of AD in adults and adolescents, these Delphi consensus recommendations provide practical guidance from a group of UK AD multidisciplinary experts with the aim of elevating the standard of care for patients with AD who currently experience high unmet need. The recommendations have been developed to be practical and easy to implement within everyday clinical practice.

Generally, participants strongly agreed to consensus statements on having a patient centric approach and intervening early. There is a large focus in the NHS of intervening early with the NHS Get It Right First Time (GIRFT) program.^31^ In AD, the effect on a patient's life course is well documented, with detrimental impacts on quality of life at home^32^ with patients particularly at risk of developing cumulative life course impairment^33^; therefore, participants felt that intervening early, especially in younger patients is critical. Furthermore, a large portion of patients with AD are in primary care, many needing more advanced treatments but are either unaware of the possibilities or unable to access secondary care services. It was suggested that primary HCPs should send requests for specialist advice through the advice and guidance service which has undergone rapid expansion since the COVID pandemic, especially within the field of dermatology (400% increase in monthly requests).^34^ Once in secondary care it is important that HCPs communicate what they hope to achieve and what may be possible given new treatment options, especially since many patients may have experienced delays in reaching the point of accessing advanced treatment, despite having moderate or severe disease. These patients tend to be less vocal about the impact of AD on their quality of life, have low expectations of what is possible with treatment^35^ and may be more willing to accept a small improvement even though larger improvements may be possible. Involving patients in treatment decisions, is widely accepted to improve empowerment and outcomes.^36^

There was broad agreement by the participants for the need of standardized assessment tools including the use of EASI and DLQI (both routinely in use within the NHS and required by NICE to assess suitability for and response to advanced therapies)^3^ with the addition of a numerical rating scale for itch, given that itch is often the most burdensome symptom for patients.^27^ Understanding how a patient is progressing against a target requires consistent measurement. It is recommended that this should be performed each time a patient is seen and documented in an accessible place. There was some discussion about whether this is required for patients who are stable on long term treatment. For these well controlled patients (not prescribed systemics, biologics and/or JAKi) consideration could be given to schemes such as patient initiated follow up (PIFU).^37^ In order to minimize the burden on HCPs of assessing disease severity consistently, completion of these should be built into practice where possible, for example making use of patient waiting times or utilizing digital tools. It is recognized that for digital tools to reach their potential, they must integrate with the NHS digital systems, however making better use of technology is in line with the proposed 10 Year Health Plan for England.^38^

Treating toward a target is a widely accepted approach globally and in Europe for the treatment of AD.^10^^,^^20^ In AD, the ultimate target should be MDA; defined as clear/almost-clear skin with no/minimal pruritus. There was acceptance from participants that for some patients, MDA may not be achievable, however the panel agreed that with the advent of new efficacious treatments and several in phase III clinical trial,^14^^,^^39^ and the implementation of patient centered treatment plans, HCPs should strive towards reaching targets of EASI 90 or absolute EASI ≤3, NRS for pruritus of ≤1, DLQI ≤3 4 to 6 months after starting a new treatment. Current NICE guidelines identify an adequate response as a 50% reduction in EASI and a 4-point reduction in DLQI at 16 weeks after starting treatment from a funding perspective,^13^ however greater improvements can be achieved for the majority of AD patients given the evolution of targeted immunomodulatory therapies in recent years. During clinic review, if targets are not reached, consideration should be given to optimizing the patient’s treatment to achieve tighter control. Participants felt it was crucial not to allow patients to remain in a sub-optimal disease state due to the significant comorbidities that typically affect a patient with AD, especially when other treatment options are available.

The main strength of this study was that participants were selected based on a wide range of specialist knowledge of AD in secondary and tertiary care from multiple geographical locations from around the UK. There is no standard size for Delphi studies^23^; however, >12 participants has previously shown diminished returns in terms of reliability.^40^ While we acknowledge the need for balance between increasing sample size, diversity of expertise and views, manageability and speed, we believe that these consensus statements will improve day to day practice and benefit patients which is sorely needed and as such we took the decision to keep the panel small and the project manageable. Furthermore, as this guidance focusses solely on the treatment and management of AD patients in secondary care, where phototherapy, biologics, JAKi and conventional immunosuppressant treatments are available, no consideration regarding primary care recommendations^41^^,^^42^ were included. However, further research may be required to determine primary care support/tools needed to triage the correct patients into secondary care.

## Conclusion

The STRIVE-AD recommendations generated by this study aim to establish bold treatment targets; using standardized and consistent measurements for disease activity; established through a shared decision-making process to optimize treatment using GIRFT principles compared with the current standard of care.

## Conflicts of interest

Gkini reports serving as an investigator and receiving honoraria, fees, and grants from NIHR studies, AbbVie, Amgen, Argenx, Pfizer, LEO Pharma, Johnson & Johnson, UCB Pharma, Novartis, Almirall, Sanofi, Pharmaserve–Lilly, Bristol Myers Squibb, Galderma, Galenica, Faran, L’Oréal, Soterius Pharma, Pierre Fabre, Beiersdorf, and Hair + Me. Alexander reports receiving honoraria and travel grants from Sanofi and AbbVie. Barfoot reports receiving honoraria and fees from Novartis, AbbVie, Galderma, and UCB, and funding for studies from LEO Pharma and UCB. Barea is the Clinical Co-lead of the Southwest London Dermatology Network and has served as a principal investigator and co-investigator in clinical research sponsored by AbbVie, LEO Pharma, UCB, and Almirall, and has received fees, honoraria, and travel grants from AbbVie, Almirall, Bristol Myers Squibb, LEO Pharma, Novartis, Pfizer, UCB, Galderma, Eli Lilly, Janssen, Biofrontera, and Sanofi. Beattie has served as a principal investigator for Eli Lilly, LEO Pharma, AbbVie, Pfizer, Almirall, Galderma, Amgen, and Sanofi, and has received fees and travel bursaries from AbbVie, Almirall, Pfizer, La Roche-Posay, and Novartis. Kreeshan reports receiving fees and honoraria from AbbVie, Almirall, Janssen, LEO Pharma, Eli Lilly, Sanofi, UCB, and Galderma, and has served as a sub-investigator for AbbVie, Janssen, and Eli Lilly. Moffitt reports receiving sponsorship and honoraria from AbbVie, Sanofi, LEO Pharma, and Galderma. Mohandas reports receiving honoraria and bursaries from AbbVie, Janssen, L’Oréal, Bristol Myers Squibb, UCB, La Roche-Posay, and Eli Lilly. Pink reports serving as an advisor, speaker, and investigator and/or receiving educational or research support from AbbVie, Pfizer, Eli Lilly, Sanofi, Galderma, Amgen, LEO Pharma, Novartis, Johnson & Johnson, UCB, Bristol Myers Squibb, and Boehringer Ingelheim. Proctor reports no conflicts of interest. Sergeant has served as an investigator or has been involved in trials with AbbVie, LEO Pharma, Novartis, and UCB, and has acted as a speaker or advisor for AbbVie, LEO Pharma, UCB, and Johnson & Johnson. Savage reports receiving fees, honoraria, and travel grants from AbbVie, Almirall, Novartis, Johnson & Johnson, Eli Lilly, UCB, Pfizer, Bristol Myers Squibb, Boehringer Ingelheim, Amgen, Medac, MoonLake, LEO Pharma, Takeda, Celltrion, Sanofi, Sandoz, Galderma, Biogen, Celgene, and Fresenius Kabi, and serves as Vice President and an elected Executive Board member of GRAPPA and as an elected committee member of the British Society of Medical Dermatology.
